# Effect of Celastrus Orbiculatus Extract on proliferation and apoptosis of human Burkitt lymphoma cells

**DOI:** 10.3389/fphar.2024.1361371

**Published:** 2024-04-03

**Authors:** Miao Zhu, Zewen Chu, Xiaojun Dai, Fan Pan, Yuanyuan Luo, Xingyi Feng, Yaqi Hu, Haibo Wang, Yanqing Liu

**Affiliations:** ^1^ Institute of Translational Medicine, Medical College, Yangzhou University, Yangzhou, China; ^2^ The Key Laboratory of Syndrome Differentiation and Treatment of Gastric Cancer of the State Administration of Traditional Chinese Medicine, Yangzhou University, Yangzhou, China; ^3^ Clinical Medical College of Yangzhou University, Yangzhou, China; ^4^ Traditional Chinese Medicine Hospital of Yangzhou, Yangzhou, China

**Keywords:** celastrus orbiculatus, lymphoma, proliferation, apoptosis, plant extract (PE)

## Abstract

The lymphoma incidence rate is on the rise, with invasive forms particularly prone to relapse following conventional treatment, posing a significant threat to human life and wellbeing. Numerous studies have shown that traditional Chinese botanical drug medicine offers promising therapeutic benefits for various malignancies, with previous experimental findings indicating that Celastrus orbiculatus extract effectively combats digestive tract tumors. However, its impact on lymphoma remains unexplored. This study aims to investigate the impact and underlying mechanisms of COE on the proliferation and apoptosis of Burkitt lymphoma cells. We diluted COE in RPMI-1640 medium to create various working concentrations and introduced it to human Burkitt lymphoma Raji and Ramos cells. To evaluate cell viability, we used the CCK-8 assay, and we observed morphological changes using HE staining. We also conducted Annexin V-PI and JC-1 staining experiments to assess apoptosis. By combining the cell cycle experiment with the EDU assay, we gained insights into the effects of COE on DNA replication in lymphoma cells. Using Western blotting, we detected alterations in apoptosis-related proteins. *In vivo* experiments revealed that following COE intervention, tumor volume decreased, survival time was prolonged, spleen size reduced, and the expression of tumor apoptosis-related proteins changed. Our findings indicate that COE effectively inhibits lymphoma cell proliferation and promotes apoptosis by regulating these apoptosis-related proteins.

## 1 Introduction

Lymphoma is a malignancy that is prevalent globally. As of 1 January 2022, it is estimated that there were 229,040 HL survivors and 845,550 NHL survivors (Miller et al., 2022). Modern medicine mainly adopts chemotherapy, radiotherapy, surgery, transplantation, biological immunity, and targeted treatment (Morris et al., 2022). Many patients are unresponsive to first-line chemotherapy or relapse after treatment remission, and some patients are not suitable for or tolerate such treatment because of their old age or systemic complications (Laribi et al., 2018; Martin-Guerrero et al., 2019; Moleti et al., 2020). Traditional Chinese medicine plant metabolites have the advantages of prolonging the survival time, improving the quality of life, and reducing adverse reactions in the treatment of lymphoma (Yuan et al., 2016). The clinical value of traditional Chinese medicine plant metabolites in the treatment of lymphoma is significant.

Celastrus orbiculatus Thunb [C. articulatus Thunb] is a traditional Chinese medicine extracted from the stem of Celastrus orbiculatus. It has the effects of dehumidification and analgesia, and is often used to treat rheumatoid arthritis, limb numbness, and other diseases (Zhu et al., 2014). Our research team has been granted a patent authorization for the ethyl acetate extract of the Celastrus orbiculatus stem, as well as its preparation and application. The patent number is 200710025343.3. At the same time, it was found that the COE can inhibit the activity of various digestive tract tumors (Jin et al., 2018; Qian et al., 2018; Lv et al., 2022; Wang et al., 2022). COE can induce apoptosis, block the cell cycle, and inhibit the proliferation of gastric cancer cells (Qian et al., 2019; Tao et al., 2021). However, it is not clear whether the COE can affect the malignant biological behavior of lymphoma. In this study, lymphoma cells RAJI and Ramos were taken as the research objects, and the effects of COE on inhibiting proliferation and promoting apoptosis of lymphoma and its specific mechanisms were studied at the molecular level through *in vitro* experiments. To investigate the molecular mechanisms underlying COE’s intervention in lymphoma progression, and establish a more robust theoretical and experimental foundation for the potential development of COE as an anti-tumor agent.

## 2 Materials and methods

### 2.1 Drugs

Celastrus orbiculatus Thunb. was purchased from Guangzhou Zhixin Pharmaceutical Co., Ltd. (Batch No.: 070510). Professor Wang Qiang of China Pharmaceutical University carried out the extraction and identification of ten plant metabolites from the ethyl acetate extract of Celastrus orbiculatus (COE). Here are ten plant metabolites extracted from the ethyl acetate fraction of Celastrus orbiculatus (COE): diterpenoid lactones derived from Prunus davidiana A (I), (5β, 8α, 9β, 10α, 16β)-16-hydroxykaurane-18-oic acid (II), salicylic acid (III), 2,4,6-trimethoxyphenol-1-O-β-D-glucoside (IV), isoquercitrin (V), quercetin-7-O-β-D-glucoside (VI), (+)-catechin (VII), vanillic acid (VIII), β-carotene (IX), and β-sitosterol (X). These plant metabolites were isolated and identified through rigorous scientific methods, ensuring their authenticity and accuracy. The main extraction method is as follows: the vine stems of Celastrus orbiculatus are crushed into powder, extracted with 95% ethanol for three times, extracted with petroleum ether for three times, and then extracted with ethyl acetate for three times. After extraction, the mixture is concentrated under reduced pressure, and the residue is vacuum freeze-dried. An appropriate concentration of dimethyl sulfoxide is added to aid in dissolution. Each Gram of the ethyl acetate extract is equivalent to 60 g of crude drug. We used high-performance liquid chromatography with diode array detection to isolate plant metabolites of different structural classes from Celastrus orbiculatus (Jiang et al., 2019; Chu et al., 2021). Vindesine Sulfate for Injection (VDS) was purchased from Hangzhou Minsheng Pharmaceutical Co., Ltd. (Batch No.: H20057028).

### 2.2 Cells and reagents

Human Burkitt lymphoma cell lines Raji and Ramos were donated by Jiangsu Key Laboratory of the Noncoding RNA Foundation and Clinical Transformation of Yangzhou University. The RPMI 1640 cell culture medium (GIBCO) and fetal bovine serum (FBS) (Hyclone Laboratories Inc.) were purchased from Abbkine Scientific (China). CCK8 was also obtained from Abbkine Scientific (China). A hematoxylin-eosin staining kit was purchased from Guangdong Baso (China). A cell cycle kit was purchased from KeyGEN BioTECH (China). The EdU staining kit, JC-1 stain kit, and Annexin V-PI kit were purchased from Shanghai Beyotime (China). Monoclonal antibodies against Bcl-2, BAX, Cleaved-caspase-3, and Bcl-xL were purchased from Cell Signaling Technology (United States). The β-actin antibody was obtained from Sigma-Aldrich (United States).

### 2.3 Cell culture

The Raji and Ramos Burkitt human lymphoma cells were grown in RPMI-1640 medium with 10% FBS and were incubated at 37°C in a humidified environment with 5% CO2.

### 2.4 Cell viability assay

The Raji and Ramos cells were seeded at a density of 5 × 103 cells per well in 96-well plates (Corning, NY, United States; Cat. no. 3599). The cells were then treated with varying concentrations of COE (0, 100, 150, 200, 250, 300, and 400 µM) and incubated overnight. They were subsequently cultured for 12 h, 24 h, and 36 h. For each well, 20 µL of CCK-8 solution was added and incubated for another 2 h. The absorbance (A) value at 450 nm was measured using an EnSpire multimode plate reader (PerkinElmer, United States). A cell viability assay was also performed on the positive control group (VDS group). The intervention concentration between COE groups was selected according to the IC 50 of COE intervention cells for 24 h. The intervention concentration for the VDS-positive group (500 ng/mL) was selected based on the VDS IC50 results (24 h).

### 2.5 Cell clustering detection and Wright’s staining

After drug treatment, cell clustering was observed under an inverted microscope. The cell suspension droplets were applied onto sterilized slides. After the slides were allowed to dry, add Wright’s staining dye solution A and dye solution B in sequence, and place the slides at room temperature for 25 min. Then, wash the dye solution carefully. The results were observed and recorded by a microscope at 1,000× magnification. The cell diameter was measured using ImageJ software.

### 2.6 Cell apoptosis assay

After drug treatment of lymphoma cells, 100×g/5 min centrifuge to discard the supernatant, 1×Binding buffer to resuspend cells and add 5 μL Annexin V-FITC incubated in dark for 15 min, then added 3 μL PI staining. Images of cell apoptosis were acquired by flow cytometer (FACSVERSE, BD Biosciences, United States) detection. The FlowJoTM 10 Software (BD Biosciences, United States) was utilized for data collection and analysis.

### 2.7 JC-1 staining

Diluted JC-1 working solution, centrifuge 100 *g*/5min to discard the supernatant after drug treatment of lymphoma cells. I added the freshly prepared JC-1 working solution, mixed it well, and incubated for 20 min. We used assay buffer to wash cells twice, and the cell suspension was detected. Images of JC-1 stain were acquired by flow cytometer (FACSVERSE, BD Biosciences, United States) detection. The FlowJoTM 10 Software (BD Biosciences, United States) was employed for data collection and analysis.

### 2.8 Cell cycle assay

After the cell suspension was added with drugs, the supernatant was discarded after centrifugation and washed twice with precooled phosphate-buffered saline (PBS). The precipitate was added to pre-cooled 75% ethanol and fixed at −20°C for 24 h. The supernatant was discarded by centrifugation and washed by PBS once, and then the supernatant was detected. Images of the cell cycle were acquired by flow cytometer detection (FACSVERSE, BD Biosciences, United States). Modfit LT 4.1 Software (Verity Software House, United States) was used for data collection and analysis. Using FSC and SSC, cell populations are delineated. Subsequently, PI-A/PI-W is employed to eliminate cell clumps. After gating, the automatic analysis function of MODFIT is utilized for cell cycle analysis. The software divides and quantifies different stages of the cell cycle, such as the G0/G1 phase, S phase, and G2/M phase, based on preset algorithms and models.

### 2.9 EDU staining

Prepare the Click reaction solution in advance according to the reagent requirements. Took out 6-well plates after dosing, added 0.5 mL of Click reaction solution into each hole, fully mixed, and incubated in dark for 30 min. After centrifugation, discard the Click reaction solution and wash it with the washing solution three times, followed by machine detection. Images of EDU were acquired by flow cytometer (FACSVERSE, BD Biosciences, United States) detection. The FlowJoTM 10 Software (BD Biosciences, United States) was employed for data collection and analysis.

### 2.10 WB analysis

After treatment, the cells were rinsed with PBS and lysed using RIPA buffer. The cells were then extracted on ice for 20 min, centrifuged at 140,00× g for 30 min, and the supernatant was collected. After boiling the protein samples, equal amounts of protein were loaded onto a sodium dodecyl sulfate-polyacrylamide gel electrophoresis (SDS-PAGE) gel for separation. The separated proteins were then transferred onto a polyvinylidene difluoride (PVDF) membrane. The membrane was blocked with 5% skimmed milk for 2 h and then probed overnight with the indicated primary antibodies, including Bcl-2, Bcl-xL, BAX, Cleaved-caspase-3, and actin. After washing, the membranes were incubated with anti-rabbit IgG and anti-mouse IgG secondary antibodies at room temperature for 2 h. Subsequently, the membranes were extensively washed and incubated using an enhanced chemiluminescent (ECL) kit (Thermo Fisher Scientific, United States). The blots were visualized using a Gel Doc XR + System (Bio-Rad, United States).

### 2.11 Nude mice

The nude mice were procured from the Center for Comparative Medical Animals of Yangzhou University. Six-week-old female nude mice were housed in specific-pathogen-free (SPF) animal facilities. The nude mice were allowed to acclimate for a week before tumor implantation. These awake mice were sacrificed by cervical dislocation. The experimenter pinched the lower back pressure of the mouse head with the thumb and forefinger of his left hand, and quickly pulled backwards by grasping the mouse tail with his right hand. The cervical vertebra of the mice was dislocated, and the spinal cord and brain marrow were disconnected. The experimental mice quickly lost consciousness and died without pain.

### 2.12 Transplanted tumors in nude mice

After a week of adaptive feeding, the lymphoma cell line Ramos was prepared in a 1 × 106/mL cell suspension containing normal saline, which was injected subcutaneously into the right armpit of mice. After 10 days of injection, the tumor bulged with naked eyes. The mice were randomly allocated into three groups, with five mice in each group: the control group, the COE group (80 mg/kg), and the COE treatment group, which received oral administration of COE for a total of 14 days (Wang et al., 2017a), while the VDS group, with a dose of 0.75 mg/kg, was administered via tail vein with VDS every 2 days for a total of 14 days (Lei et al., 2018). Commencing from the intervention, the fluorescence intensity at the tumor site in mice was assessed weekly utilizing an animal live imaging system (PerkinElmer, Waltham, MA, United States). This fluorescence intensity serves as an indicator of the tumor’s size and metastatic location. The experimental animal ethics committee of Yangzhou University granted approval for this study (SYXK Su 2022-0044), and we conform to the guidelines of the Ethics Committee for Animal Research (NIH publication #85-23).

### 2.13 Immunohistochemistry

Mice were sacrificed after intervention, the tumor body and spleen of mice were weighed, and a part of tumor tissue was cut and added with lysate to extract protein. The remaining tumor tissue was fixed in formalin, embedded in paraffin, and then sliced into 5-micron sections. The sections were dewaxed and rehydrated. After the antigen was exposed, the antibody was used for immunostaining, then the second antibody was incubated, and the film was dehydrated and sealed after staining.

### 2.14 Statistical analysis

The data are presented as the means ± standard deviation, which were determined through one-way analysis of variance (ANOVA) followed by a Bonferroni *post hoc* test. *p < 0.05 was considered significant. The data analysis was conducted using IBM SPSS Statistics 24.0 (IBM, New York, United States).

## 3 Results

### 3.1 Effects of COE on the viability of Raji and Ramos cells

**FIGURE 1:**
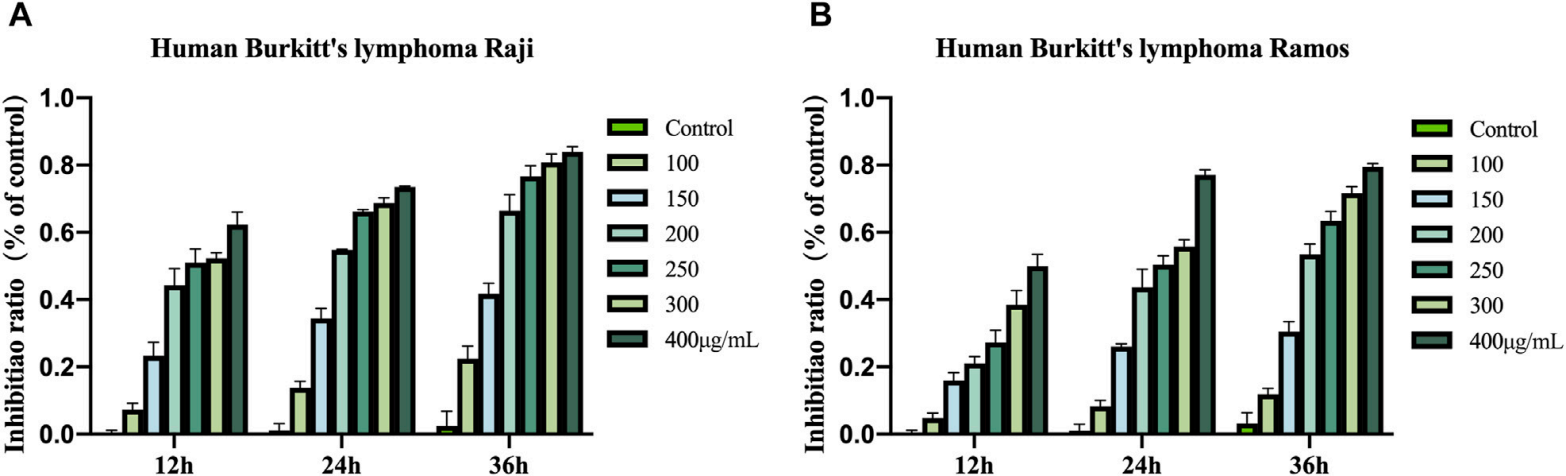
**(A)** Growth inhibitory effects of COE on Raji. **(B)** Growth inhibitory effects of COE on Ramos.

The CCK-8 assay revealed that Raji and Ramos cells treated with COE exhibited concentration- and time-dependent inhibition compared to the control group (Figures 1A,B).

### 3.2 Effects of COE on morphology in human lymphoma cells

**FIGURE 2:**
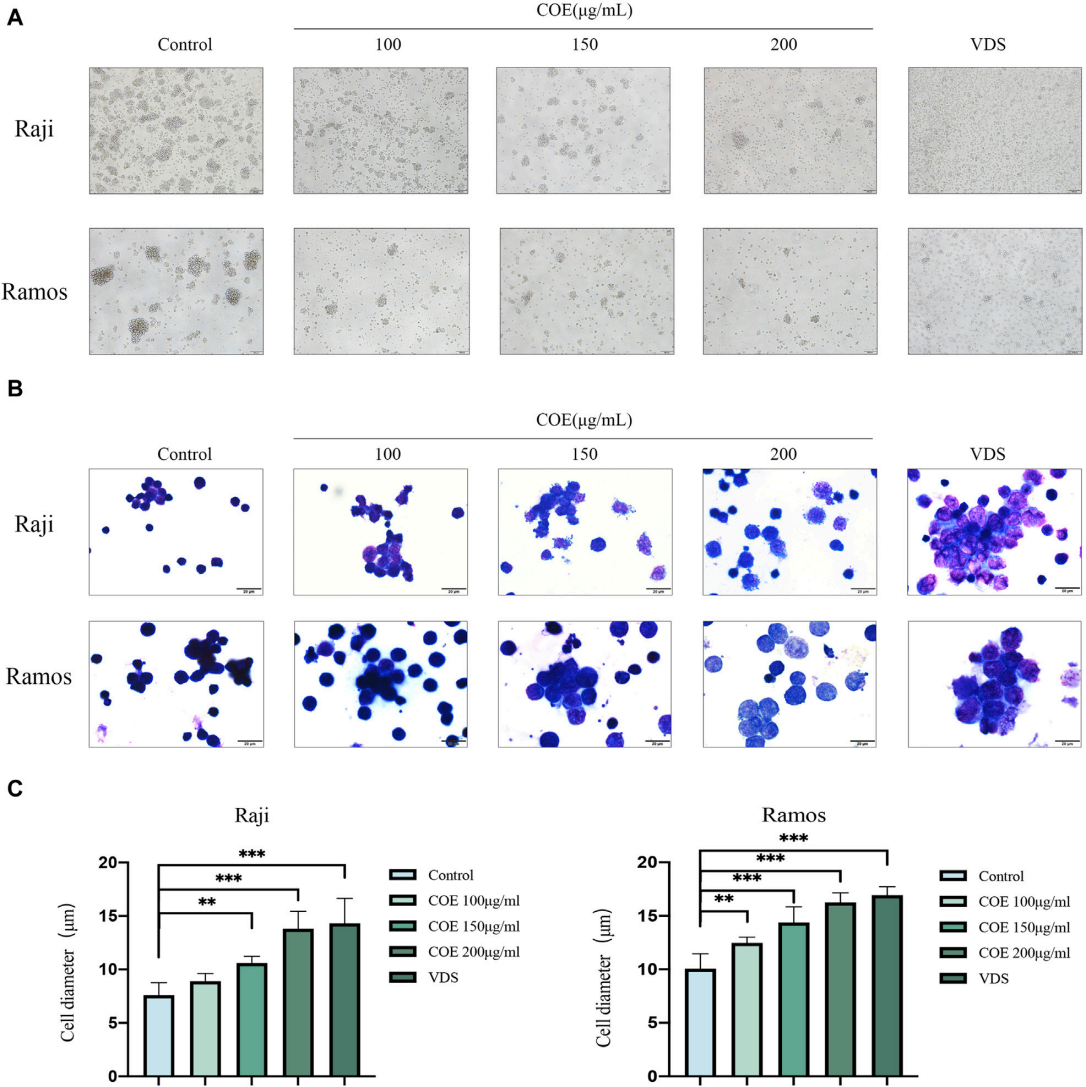
**(A)** The lymphoma cell mass showed a reduction in size after the intervention, and the cells appeared irregularly rounded. Images were captured using an inverted microscope at ×100 magnification, and all scale bars in the figure represent 200 µm. **(B)** The HE staining revealed that following the intervention, the lymphoma cells exhibited an increase in volume and became more transparent. Additionally, some of the cell membranes appeared incomplete. Images were captured using a microscope at 1,000× magnification, and all scale bars in the figure represent 20 µm. **(C)** The changes in lymphoma cell diameter after 24 hours of COE treatment were statistically analyzed. After the treatment, the diameter of lymphoma cells increased, and the results were statistically significant. ^**^ *p*<0.01, ^***^ *p*<0.001.

In order to study the inhibitory effect of COE on human lymphoma cells, it was observed under the microscope that the number of clusters of lymphoma cells decreased after the intervention (Figure 2A). Subsequently, the lymphoma cells were stained with a dye solution. Under the microscope, the cell morphology was irregular, and the cell volume significantly increased. These results showed that COE could affect the morphology of lymphoma cells (Figures 2B,C).

### 3.3 Effects of COE on apoptosis in human lymphoma cells

**FIGURE 3:**
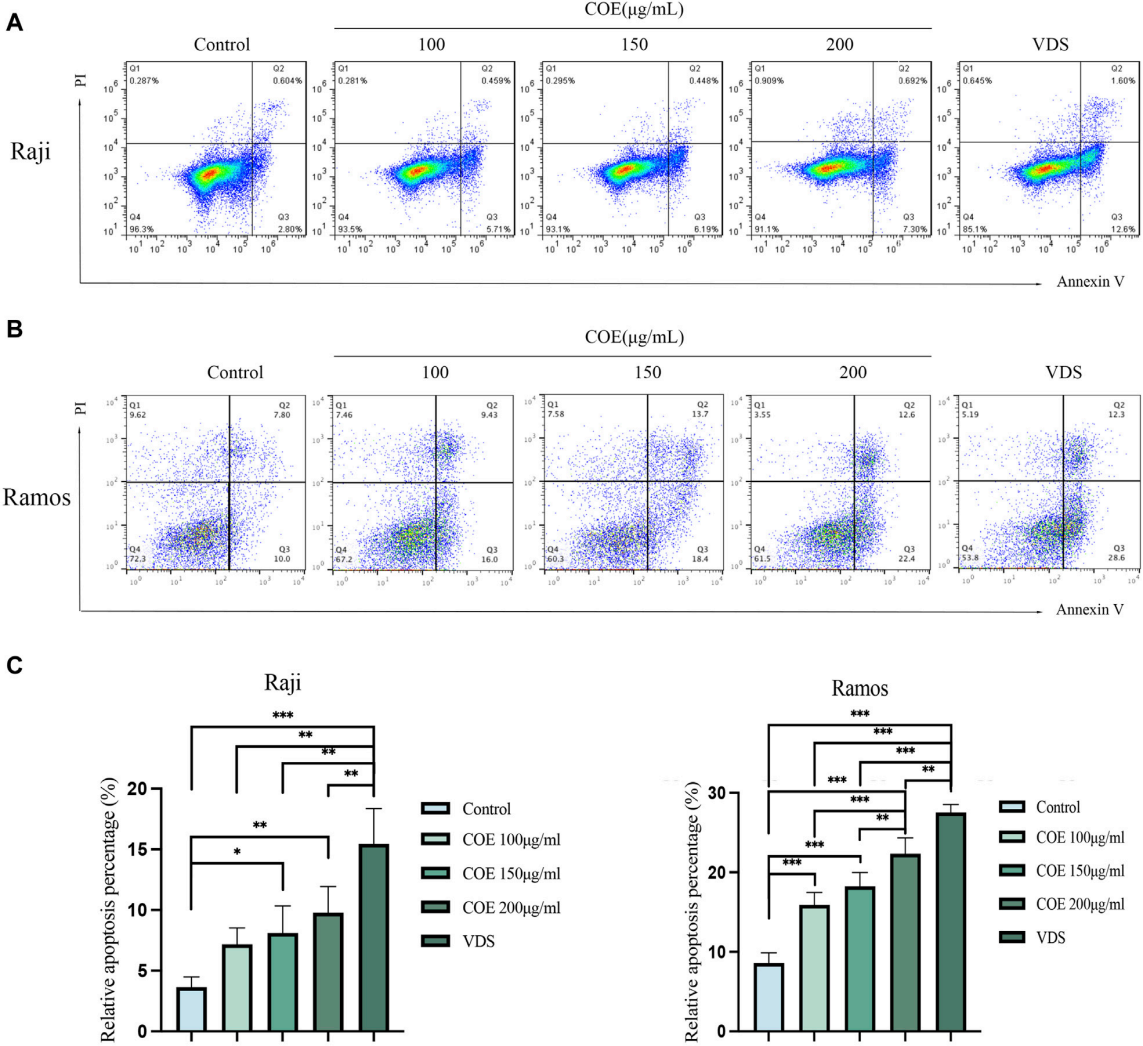
**(A, B)** COE treatment for 24 h promoted the apoptosis of Raji and Ramos cells, **(C)** Figure C provides the statistical analysis of the apoptosis rate. It is evident that the apoptosis rate in the COE 150 μg/mL and 200 μg/mL groups was significantly higher compared to the control group. Notably, the VDS group exhibited the highest apoptosis rate among all the groups. The results were statistically significant. *p < 0.05, **p < 0.01, ***p < 0.001.

In order to further study the effect of COE on the apoptosis of lymphoma cells *in vitro*, the Annexin V-PI apoptosis staining experiment was carried out. The result showed that, compared with the control group, the proportion of apoptotic cells in Raji and Ramos lymphoma cell groups treated with COE increased (Figures 3A,B). The results were statistically significant (Figure 3C).

### 3.4 Effects of COE on changes in mitochondrial membrane potential in human lymphoma cells

**FIGURE 4:**
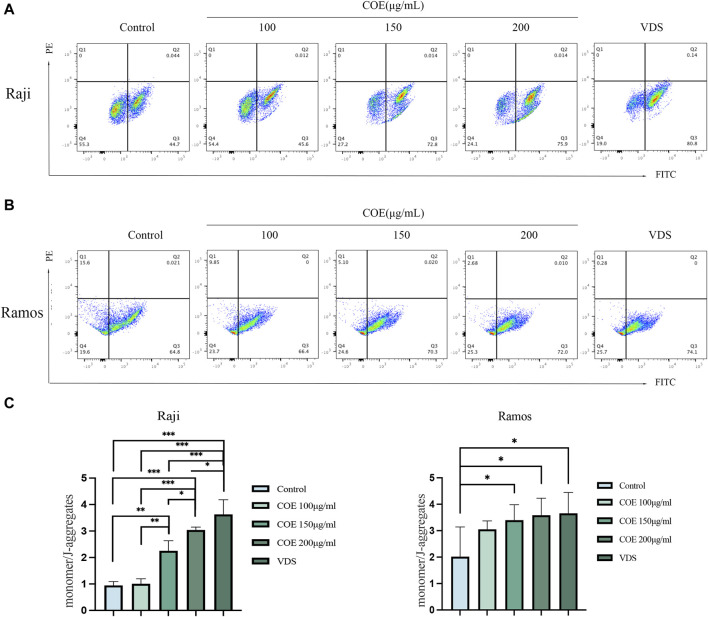
**(A, B)** Compared with the control group, JC-1 staining showed that the mitochondrial membrane potential of Raji and Ramos lymphoma cells treated with COE was downregulated, which revealed that the ratio of JC-1 monomer/aggregates was significantly increased. **(C)** The ratio of monomers to aggregates in each group is shown in the figure below. *p < 0.05, **p < 0.01, ***p < 0.001.

The JC-1 assay results indicated that in the Raji and Ramos cell lines, the COE and VDS intervention led to a decrease in the red fluorescence intensity of JC-1 aggregates, a significant increase in the green fluorescence of JC-1 monomers, and a reduction in mitochondrial membrane potential compared to the control group (Figures 4A,B). The results were statistically significant (Figure 4C).

### 3.5 Effects of COE on cell cycle and proliferation in human lymphoma cells

**FIGURE 5:**
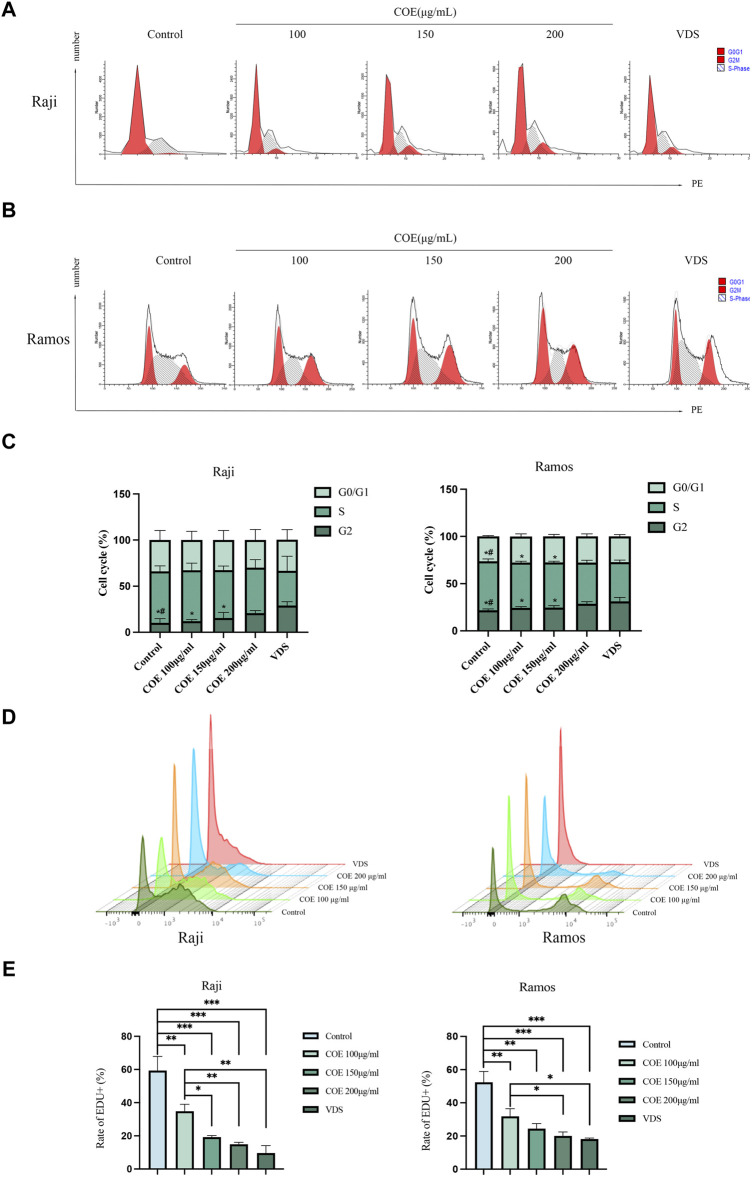
**(A, B)** The cell cycles of Raji and Ramos after COE intervention were detected by flow cytometry. **(C)** When compared to the control group, the G2 phase population was significantly higher in the VDS and COE 200 μg/mL groups. When compared to the VDS group, the difference was statistically significant (*p < 0.05). When compared to the COE 200 μg/mL group, there was also a significant difference (#*p* < 0.05). **(D)** The ratio of EDU in Raji and Ramos cells after COE intervention was detected by flow cytometry. **(E)** Compared with the control group, the ratio of EDU in the VDS and COE intervention groups was significantly decreased. *p < 0.05, **p < 0.01, ***p < 0.001.

In order to further observe the inhibitory effect of COE on lymphoma cells, we detected the cell cycle and carried out EDU assays to prove the inhibition of COE on cell proliferation. Raji and Ramos cells showed that the cell cycle changed with the concentration of the intervention drugs (Figures 5A,B). The G2 phase of lymphoma cells increased with the increase in COE concentration (Figure 5C). The EDU assays revealed that the cell DNA replication activity decreased after drug intervention (Figure 5D), and the lymphoma cell activity continued to decrease with the increase in COE concentration (Figure 5E).

### 3.6 Effects of COE on the expression of apoptosis-related proteins in human lymphoma cells

**FIGURE 6:**
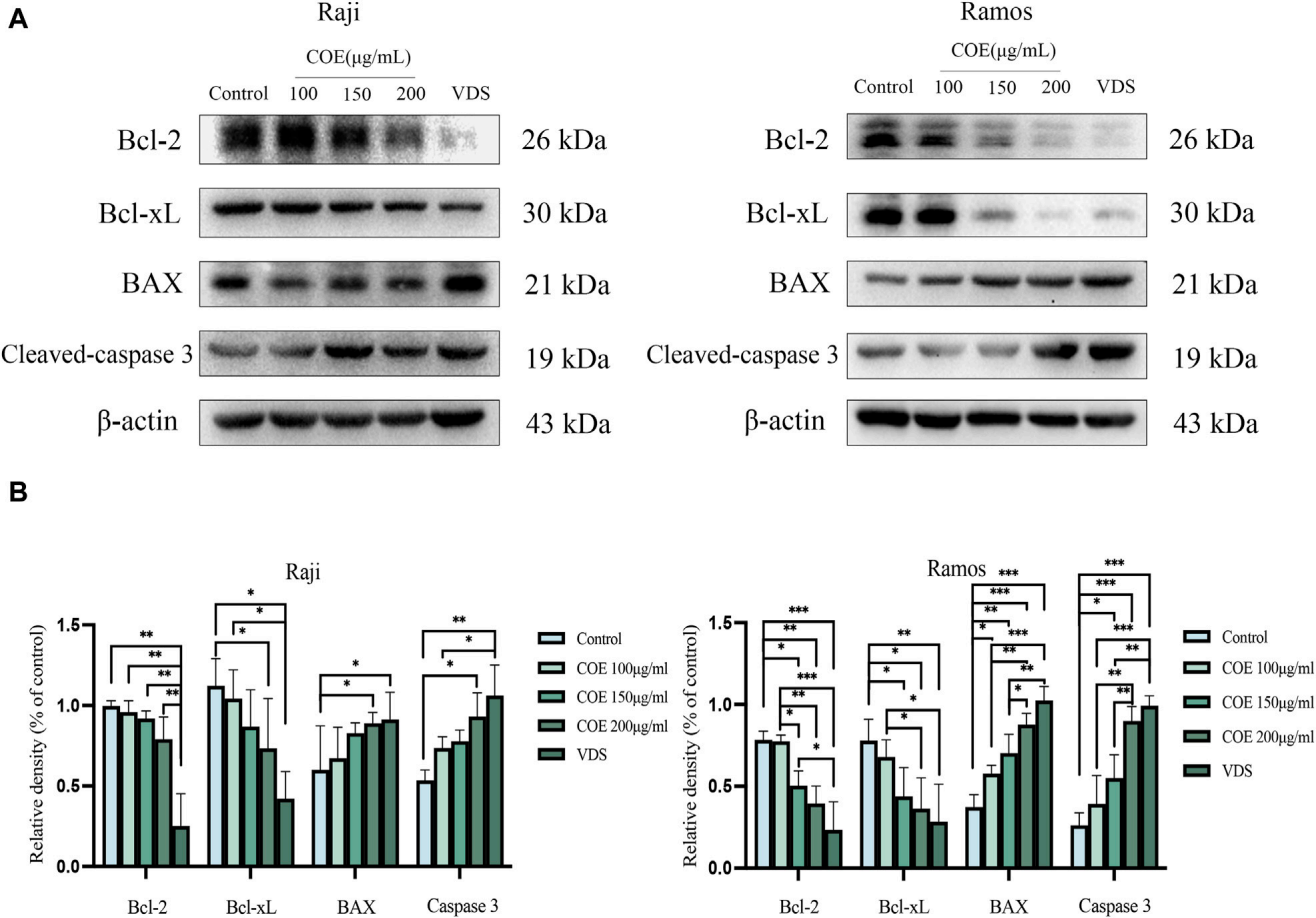
**(A)** Apoptosis protein levels of Bcl-2, Bcl-xL, BAX, Cleavd-caspase-3, and β-actin in Raji and Ramos cells were shown after COE intervention. **(B)** In comparison to the control group, the expression of Bcl-xL protein was significantly lower in the Raji and Ramos cells treated with VDS and COE 200 μg/mL. Conversely, the levels of BAX and Cleaved-caspase-3 proteins were significantly higher in these groups. *p < 0.05, **p < 0.01, ***p < 0.001.

Raji and Ramos cells were treated with COE for 24 h. In Figure 6, it is evident that the protein expression of BAX and Cleaved-caspase-3 is upregulated, while the expression of Bcl-2 and Bcl-xL is downregulated. (Figures 6A, B).

### 3.7 COE’s antitumor effect on lymphoma-bearing mice *in vivo*

**FIGURE 7:**
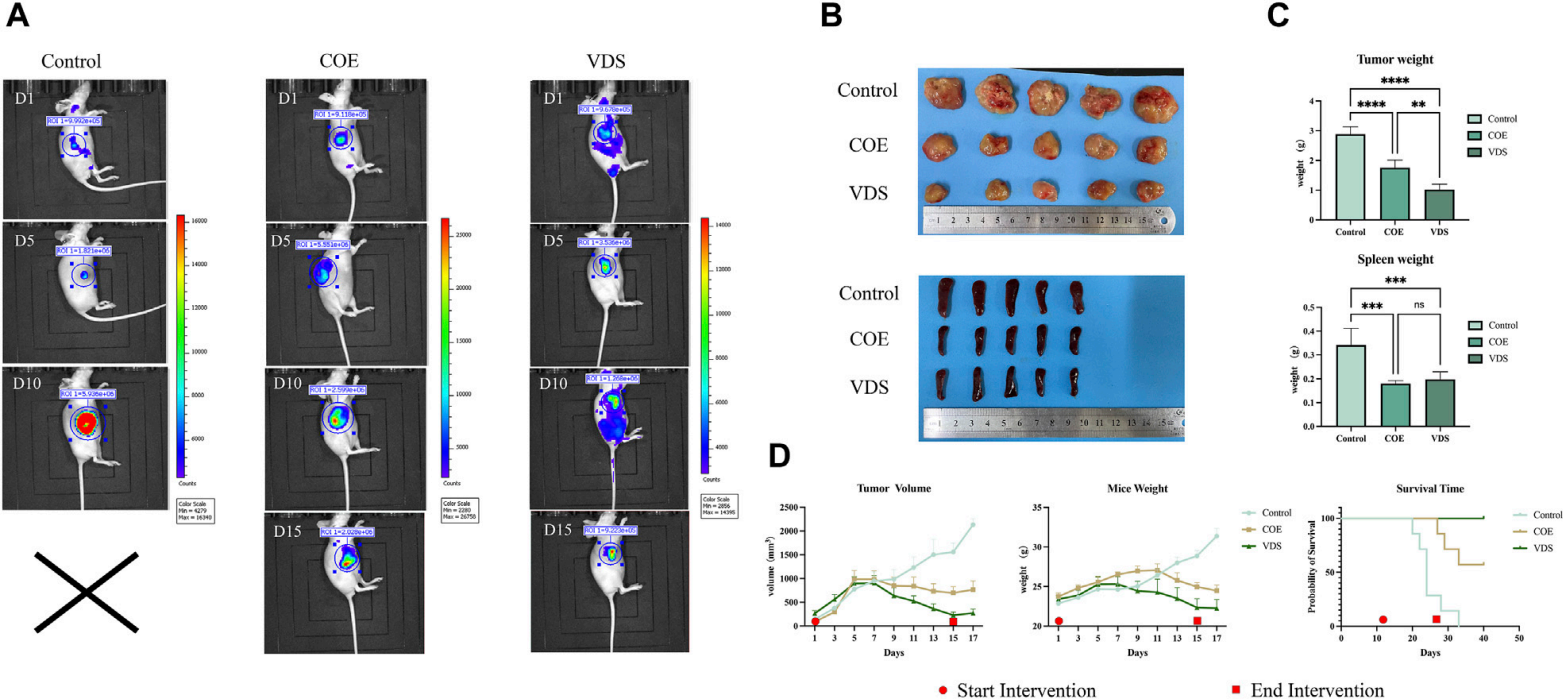
**(A)** Changes of *in vivo* imaging of mice in the COE, VDS, and control groups after intervention. **(B)** General picture of tumor body and spleen in COE, VDS, and control groups. **(C)** Compared with the control group, the tumor size in the COE group and the VDS group was smaller, and the degree of spleen swelling was lower. **(D)** Compared with the control group, the tumor volume of mice in the COE group decreased, and the survival time of mice was prolonged. *p < 0.05, **p < 0.01, ***p < 0.001.

**FIGURE 8:**
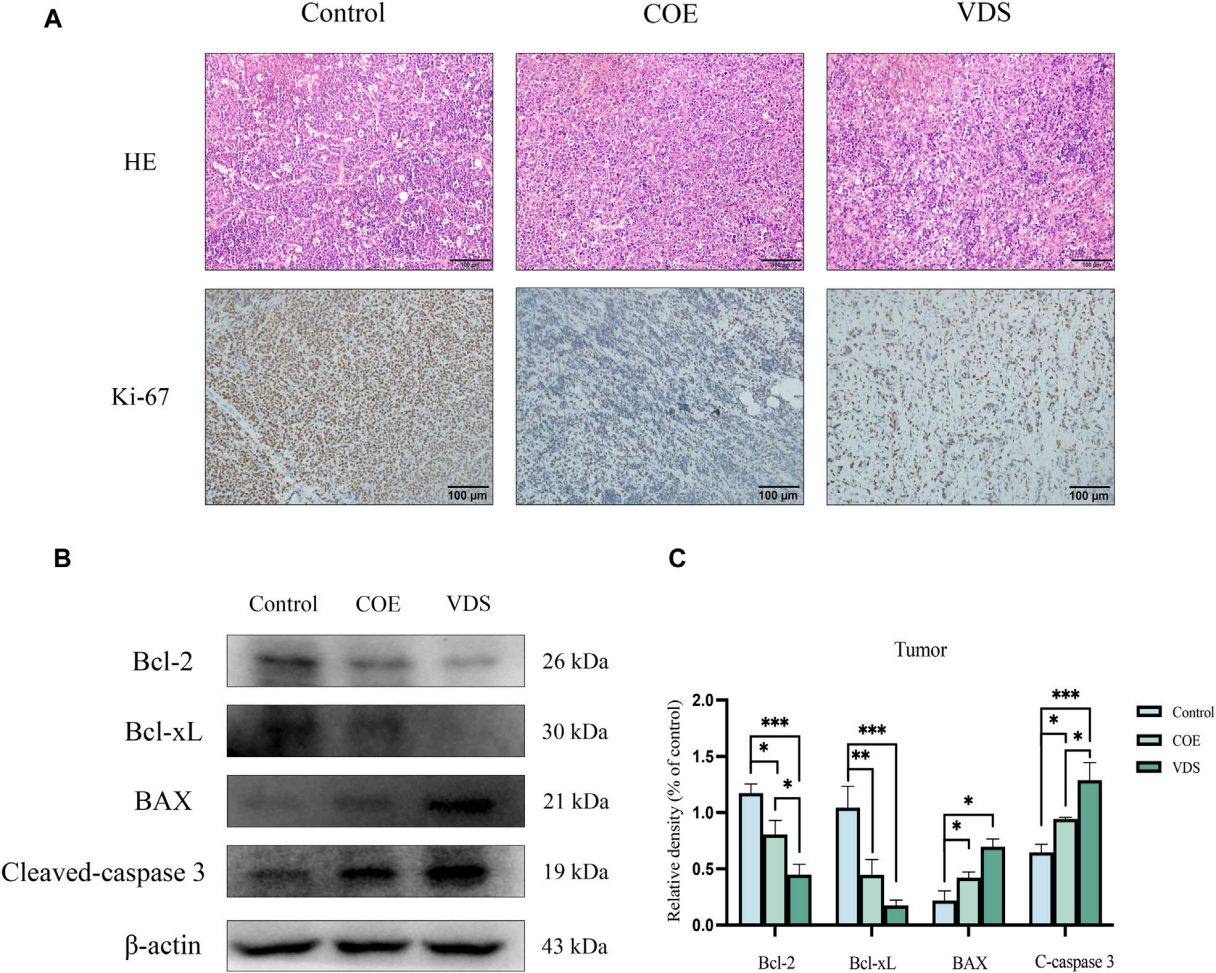
**(A)** After COE intervention, tumor necrosis, tumor vascular atrophy, and Ki-67 expression decreased. **(B, C)** Compared with the control group, the levels of apoptotic proteins Bcl-xL and Bcl-2 in the VDS and COE groups were significantly decreased, while the levels of BAX and Cleavd-Caspase three were significantly increased. *p < 0.05, **p < 0.01, ***p < 0.001.

COE intervened in lymphoma-bearing mice for 14 days, and compared with the control group, the tumor size was reduced, the survival time was prolonged, and the spleen size was reduced (Figure 7). Compared with the control group, the tumor tissue is necrotic, the blood vessels in the tumor are atrophied, the protein expressions of BAX and Cleaved-Caspase-3 in the tumor are increased, and the protein expressions of Bcl-2 and Bcl-xL are decreased (Figure 8).

## 4 Discussion

In addition to chemotherapy and radiotherapy, immunotherapy, and molecular targeted therapy, the treatment of lymphoma with high efficiency and low toxicity in traditional Chinese medicine therapy has gradually entered clinical work. Celastrus orbiculatus is a deciduous vine-like shrub of Celastrus orbiculatus of Celastraceae, and its rhizome can be extracted with medicinal effects such as dispelling wind, removing dampness, dredging channels, relieving pain, promoting blood circulation, and detoxicating (Xu et al., 2022). In our previous research, we found that COE can effectively inhibit the proliferation of tumor cells, such as gastric cancer, lung cancer, and esophageal cancer. Additionally, COE promotes apoptosis by regulating cell proliferation, migration, invasion, apoptosis, autophagy, and cycle distribution. These findings suggest that COE may be a potential therapeutic agent for the treatment of various cancers (Qian et al., 2012; Zhang et al., 2012; Li et al., 2014; Yang et al., 2016; Wang et al., 2017a; Wang et al., 2018; Jang et al., 2020). In this study, we demonstrated that COE could also interfere with Burkitt lymphoma cells and inhibit their proliferation.

Burkitt’s lymphoma is a highly aggressive B-cell lymphoma derived from the germinal center of lymphoid follicles, with high invasiveness and a poor prognosis (Crombie and Lacasce, 2021; Evens et al., 2021). Extracts from traditional Chinese medicinal plants have been proven to have good therapeutic effects on Burkitt’s lymphoma (Kong et al., 2018; Ni et al., 2018). Raji and Ramos, human Burkitt’s lymphoma in the logarithmic phase, were selected and treated with COE at different doses. The results showed that COE could inhibit the proliferation of human lymphoma cells and induce the apoptosis of lymphoma in a dose-dependent and time-dependent manner.

After intervention by COE, Raji and Ramos cells became more transparent and pale-stained, and the whole cell expanded. We speculated that the COE might be responsible for the damage of the sodium pump on the cell membrane, dysfunction of cell membrane transport, and increased intracellular water content, resulting in cell edema (Panickar et al., 2015). To confirm the presence of apoptosis in the swollen cells, the Annexin V-PI double staining experiment was conducted, which revealed a significant increase in the number of early apoptotic cells. Apoptosis is frequently associated with the disruption of mitochondrial transmembrane potential, which is widely recognized as one of the earliest events in the apoptotic process (Lu et al., 2018). The observed decrease in mitochondrial membrane potential following COE intervention further supports its role in promoting apoptosis in lymphoma cells. Additional studies have demonstrated that COE can induce cell cycle arrest in the G2/M phase and reduce the proportion of cells in the S phase, which is involved in DNA and histone synthesis. The EDU experiment further corroborates these findings by showing a decrease in DNA replication levels in lymphoma cells after COE intervention.

The regulation of apoptosis is complex and involves numerous apoptotic genes, including both anti-apoptotic genes like Bcl-2 and Bcl-xL as well as pro-apoptotic genes like BAX and Caspase 3. Bcl-2, a protein that promotes cell survival, inhibits the release of Cytochrome C from the mitochondria into the cytoplasm, thus preventing apoptosis from occurring (Fang et al., 1998). The Bcl-2 family protein Bcl-xL can interact with other apoptotic proteins to block their apoptotic effects. It also plays a crucial role in maintaining the normal membrane state of mitochondria under stress conditions by directly forming pores in the mitochondrial outer membrane (Ding et al., 2014; Pécot et al., 2016). In contrast, BAX is a crucial pro-apoptotic gene. Its overexpression can counteract the protective effects of Bcl-2, rendering cells more prone to death. The balance between BAX and Bcl-2 proteins is a critical determinant of the strength of apoptosis inhibition (Zheng et al., 2016). Another key pro-apoptotic protein is Caspase 3, the most critical terminal shearing enzyme involved in the execution of apoptosis (Rennier and Ji, 2015). COE reduced the expression levels of Bcl-2, Bcl-xL, and the PI3K/Akt/mTOR/p70s6k signaling pathway proteins, while simultaneously enhancing the levels of Bax and caspase proteins in gastric cancer cells (Wang et al., 2017b). In this study, we examined the impact of COE on apoptotic proteins in Burkitt’s lymphoma cells. Our findings indicate that COE downregulates the expression of anti-apoptotic proteins Bcl-2 and Bcl-xL, while upregulating pro-apoptotic proteins BAX and Caspase 3. This shift promotes apoptosis in lymphoma cells. To further investigate the underlying mechanism of COE-induced apoptosis, we plan to explore how COE affects cell signaling pathways involved in apoptosis. Additional experiments, including Annexin V-PI double staining and JC-1 staining, have provided further evidence of the growth-inhibitory and pro-apoptotic effects of COE on lymphoma cells. These findings suggest that COE may represent a promising therapeutic approach for the treatment of lymphoma.

## 5 Results

In summary, this study has shown that COE exerts an impact on the proliferation and apoptosis of Burkitt lymphoma cells. The underlying mechanism may involve the regulation of apoptotic proteins, with upregulation of pro-apoptotic proteins BAX and Cleaved-caspase3 and downregulation of anti-apoptotic proteins Bcl-2 and Bcl-xL. These changes lead to the inhibition of cell proliferation and promotion of apoptosis. These findings suggest that COE may offer a potential therapeutic strategy for the treatment of Burkitt lymphoma. This study offers a novel perspective for the investigation of the mechanism underlying COE’s therapeutic effects in lymphoma treatment. Additionally, our findings provide a more robust theoretical and experimental foundation for future *in vivo* research. The results presented here further support the development of COE as a potential novel anti-tumor agent.

## Data Availability

The raw data supporting the conclusions of this article will be made available by the authors, without undue reservation.
